# A Review of the Role of Estrogens in Olfaction, Sleep and Glymphatic Functionality in Relation to Sex Disparity in Alzheimer’s Disease

**DOI:** 10.1177/15333175241272025

**Published:** 2024-08-08

**Authors:** Anupa Ekanayake, Senal Peiris, Biyar Ahmed, Sangam Kanekar, Cooper Grove, Deepak Kalra, Paul Eslinger, Qing Yang, Prasanna Karunanayaka

**Affiliations:** 1 Department of Radiology, 12310 Penn State University College of Medicine, Hershey, PA, USA; 2 186703 Grodno State Medical University, Grodno, Belarus; 3 Department of Neurology, 12310 Penn State University College of Medicine, Hershey, PA, USA; 4 Department of Neurosurgery, 12310 Penn State University College of Medicine, Hershey, PA, USA

**Keywords:** alzheimer’s disease, estrogen, olfactory system, menopause, sleep, glymphatic system, sex disparity

## Abstract

Several risk factors contribute to the development of Alzheimer’s disease (AD), including genetics, metabolic health, cardiovascular history, and diet. It has been observed that women appear to face a higher risk of developing AD. Among the various hypotheses surrounding the gender disparity in AD, one pertains to the potential neuroprotective properties of estrogen. Compared to men, women are believed to be more susceptible to neuropathology due to the significant decline in circulating estrogen levels following menopause. Studies have shown, however, that estrogen replacement therapies in post-menopausal women do not consistently reduce the risk of AD. While menopause and estrogen levels are potential factors in the elevated incidence rates of AD among women, this review highlights the possible roles estrogen has in other pathways that may also contribute to the sex disparity observed in AD such as olfaction, sleep, and glymphatic functionality.

## Introduction

Alzheimer’s disease (AD) is a progressive neurodegenerative disorder that ultimately leads to dementia, resulting in a loss of independent functioning. While there is no clear etiology for AD, distinct biomarkers such as β-amyloid depositions, tau tangles, and brain atrophy have been identified for diagnosing AD and related dementias (ADRDs) that hinder autonomy and negatively impact well-being and sense of purpose in older age.^1^ The number of Americans with ADRD will continue to increase in the coming years, from an estimated 6.7 million today to a projected 13.8 million by 2060. In addition to those diagnosed with ADRD, 16.6% of individuals aged 65 and older are estimated to have symptoms of mild cognitive impairment (MCI) that may constitute a preclinical stage of AD.

The risk for developing AD increases with age and neuropathologically it is identified with certain types of protein accumulation such as amyloid plaques and neurofibrillary tangles.^2,3^ Amyloid precursor protein (APP), a transmembrane protein, is the source of beta-amyloid peptide; the proteases known as alpha, beta, and gamma-secretases separate beta-amyloid peptide from APP. Typically, either alpha-secretase or beta-secretase cleave APP, and the minute byproduct fragments of this process are not harmful to neurons. On the other hand, the 42 amino acid peptides (beta-amyloid 42) are produced via successive cleavages of beta and gamma-secretases. Amyloid aggregates, capable of causing neuronal damage are produced when beta-amyloid 42 levels are elevated; beta-amyloid 42 favors aggregated fibrillary amyloid protein production over typical APP breakdown. Concurrently, tau protein forms fibrillary intracytoplasmic aggregates in neurons known as neurofibrillary tangles, disrupting tau proteins’ primary task of stabilizing axonal microtubules. For intracellular transport, microtubules are crucial. They are found along neuronal axons, where tau protein maintains the integrity of the microtubule assembly. Tau becomes hyperphosphorylated in AD as a result of extracellular beta-amyloid aggregation, leading to the production of aggregated tau. Neurofibrillary tangles, which are formed by tau aggregation, are twisted pairs of helical filaments; within neurons, tau aggregates build up, leading to further neurotoxicity.

The lack of an established AD etiology makes the identification of risk factors crucial for the prevention and early diagnosis of AD difficult. Several well-established risk factors for AD exist, including age, genetics (specifically APOE-e4 status), metabolic health, cardiovascular history, diet, and level of formal education, among others. ADRDs can also reflect substantial health disparities encompassing economic inequalities and minority groups.^4^

**Figure 1.**
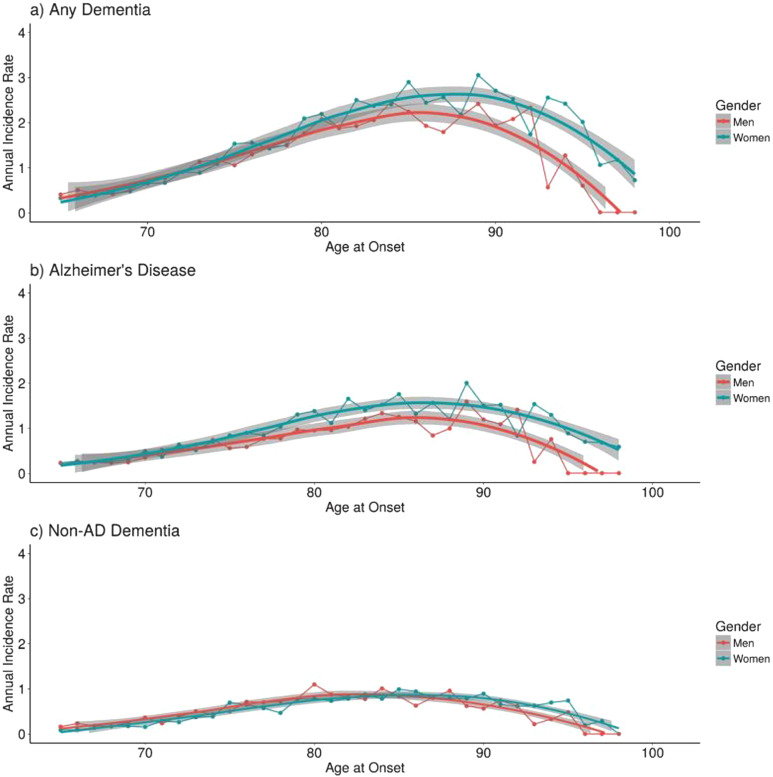
Incidence density rates of a) any dementia, b) Alzheimer’s disease, and c) non-AD dementia per 1000 person-years in Swedish men and women twins across late adulthood. Loess smoothing lines (with 95% confidence interval) were fit using nonparametric local polynomial regression fitting methods. Note: Beam, C. R., Kaneshiro, C., Jang, J. Y., Reynolds, C. A., Pedersen, N. L., & Gatz, M. (2018). Differences Between Women and Men in Incidence Rates of Dementia and Alzheimer’s Disease. Journal of Alzheimer’s Disease, 64(4), 1077–1083. https://doi.org/10.3233/JAD-180141.

Notably, women have shown a higher incidence rate of AD compared to men for reasons that are unclear.^2,5,6^ For example, in the United States, 4.1 million women over the age of 65 have been diagnosed with AD as opposed to 2.6 million men in the same age group.^1^ This gender discrepancy has been investigated on a global scale, yielding somewhat mixed results. For example, a Swedish study demonstrated that the annual AD incidence rates between men and women were not significantly different until the age of 80, after which they diverged (Figure 1).^7^ These inconclusive findings have piqued scientific interest in exploring potential biological systems between males and females that could influence susceptibility to AD.

Differences in longevity between the two genders can be a potential factor leading to a discrepancy in the diagnosis of AD. If the group with lower lifespan is due to other causes of mortality, then they may not have been diagnosed with AD during its preclinical stages. Olfactory deficits have been identified as a potential early symptom in preclinical AD, allowing them to be used as a diagnostic test of great value. Though loss of olfactory function can be used as a disease marker in AD, it must be calibrated between the two genders, as females are known to have higher olfactory abilities compared to males, and also the age-related changes in olfactory-related brain structures may differ between the two genders.

Estrogen can play a prominent role in the observed gender disparity in AD. It is known to affect many biological systems. For example, estrogen changes can potentially accelerate age-related decline in brain function due to its association with systemic and central nervous system inflammation.^8^ Changes in cognition between males and females is also reflected in the transition of mild cognitive impairment (MCI) to AD; for instance, females are at a higher risk of converting irrespective of age and education level.^9,10^

Changes in the biological functions of estrogen due to its withdrawal in midlife, therefore, seems to increase multiple AD risk factors. It has the potential to shed light on why women are at a lower risk for AD before menopause but at a significantly higher risk after menopause.^10^ Estrogen has a myriad of effects. However, this review specifically focuses on answering the question: does estrogen play a role in olfaction, in sleep, and in glymphatic function? All three of these factors have been associated with AD and such information as this review presents can provide opportunities for early intervention.^11^

## Methodology

### Search Criteria

We performed literature searches following the Preferred Reporting Items for Systematic Reviews and Meta-Analyses guidelines. Initially, the PubMed and Google Scholar databases were searched with the following keywords: “estrogen*”, “sex”, “women”, “female”, combined with “dement*”, “Alzheimer*”, “cogni*”, “longevity” and “MCI”. The functionality of estrogen in humans is multifaceted, and can influence many biological processes. We, therefore, focused on topics that dealt with how variations in estrogen during menopause influence olfaction, sleep and glymphatic functionality, and how hormone replacement therapy might ameliorate menopause-related symptoms, potentially reducing the risk for AD pathology in women.

We excluded studies that focused on gender disparity in other neurodegenerative diseases such as Parkinson’s and multiple sclerosis. Research that only focused on males and other steroid hormones, such as progesterone, again with regard to gender disparity in AD, were also excluded.

Given the broad nature of this review, we expanded our initial search, considering the following observations:

• Gender disparity in AD has been linked to longevity differences between genders.
• Relationships between estrogen, sleep, glymphatic system, AD and olfaction.

Fluctuating estrogen levels during menopause can affect olfaction, which is a preclinical symptom of AD. Estrogen level changes impact sleep, which is closely tied to the glymphatic system. The pathophysiology of AD is closely related to the glymphatic functionality which removes toxic metabolites from the brain parenchyma. Therefore, the expanded PubMed and Google Scholar database searches included the following additional keywords: “olfaction*”, “sleep” and “glymphatic system”. The literature included in this review provided us with information that would help determine the role of estrogens in olfaction, sleep and glymphatic functionality in relation to AD. Given the exploratory nature of this review, we included studies discerning processes for identifying vulnerable or at-risk menopausal women for AD, instead of studies taking AD or MCI only as a starting point. There was, however, significant overlap between topics. Our scoping review focused on the respective biological systems at the human-animal research interface. The following critique presents an overview of these major functions, highlighting the potential role of estrogen in MCI and AD.^12^

## Current Theories for Sex Disparity in AD

### Longevity

Compared to men, women have longer life expectancies and therefore lower mortality rates. Additionally, data support that men are more likely to develop MCI than women.^13^ Thus, it can be hypothesized that the higher incidence rates of AD in women may be attributed to their longevity, enabling them to reach an age at which risk for AD diagnosis becomes substantially higher. In other words, men could be passing away from other causes before AD symptoms fully manifest, resulting in forestalled AD diagnosis in men. Figure 1 supports this hypothesis as the higher rates of AD diagnoses in women could be correlated with greater survival rates, specifically, above the age of 80 with the previously mentioned Swedish cohort.^7^ However, the US Alzheimer’s Association reported that AD is more frequently diagnosed in women over the age of 65, with 12% of women in this age range compared to 9% of men.^1^

There are other approaches to testing this longevity hypothesis. For example, investigations could focus on comparing postmortem diagnostic biomarkers of AD (i.e., amyloid plaques and neurofibrillary tangles). If this hypothesis is supported, it would suggest that more men have undiagnosed AD neuropathology, which could help explain this sex disparity. Alternatively, a more robust screening process and improved diagnostic tools could be employed to ascertain whether AD is indeed underdiagnosed in men. One potential avenue for early AD detection involves exploring the connections between olfaction and neurodegeneration.^14,15^

### Olfaction

Olfactory deficits consistently have been identified as predictors of increased mortality and a heightened risk for dementia.^16,17^ These deficits frequently can precede symptoms of memory and cognitive decline in AD.^16,18^ In particular, odor-detection and odor-identification are significantly impaired in early AD patients compared to age-matched controls.^16,17^ Longitudinal studies of AD have further indicated that early olfactory deficits are correlated with the severity of dementia.^17^ Patients with “questionable” AD or “at-risk” AD also demonstrate a significantly higher olfactory sensory detection threshold.^16^ Odor identification tests have been proven to be effective in early detection of amnestic MCI disorders, which are considered to be a high-risk phase for progression to dementia.^16^ Moreover, it has been reported that elderly individuals with intact olfactory function are less likely to develop AD or dementia over a 5-year period.^16,18^ It is commonly accepted that women have better sense of smell when compared to men.^19^ In standardized olfactory tasks such as the University of Pennsylvania Smell Identification Test (UPSIT), women have been shown to consistently outperform men at older ages.^20^

It is important to note that there is a similar level of olfactory function loss with age in both sexes, suggesting a higher olfactory baseline for women that must be accounted for when assessing for olfactory deficits. A recent study, however, indicated that age-related volume losses in the olfactory cortex starts earlier in women compared to men. Thus, volume changes in olfactory-related brain regions in older subjects could serve as potential proxies for the increased risk for neurodegeneration.^21^ While these findings provide compelling evidence for a relationship between olfactory deficits and AD onset/progression, the precise nature of this interdependency requires further investigation. Once a quantifiable link between the two is more firmly established, it may become possible to identify individuals with an elevated risk of AD before the onset of neurological deficits. Such advancements could contribute to unraveling the sex disparities observed in AD.

## Estrogen’s Role in Alzheimer’s Pathology

**Figure 2.**
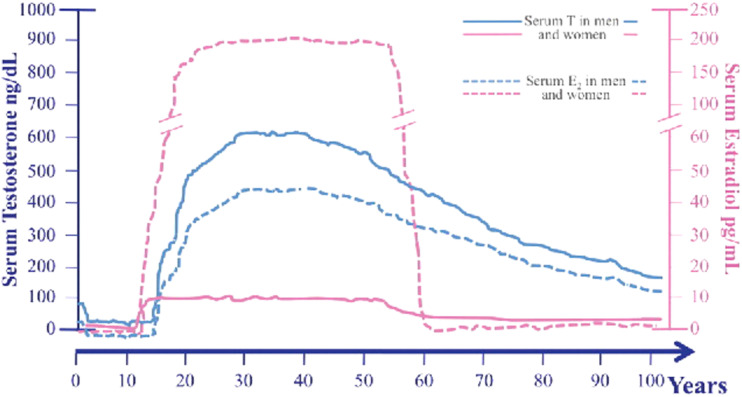
Serum levels of both testosterone (T) and estradiol (E2) across the lifespan in men and women. Serum E2 levels of premenopausal women are represented as the mean of E2 measured during the different phases of the menstrual cycle; T: testosterone; E2: estradiol. Note: Decaroli, M. C., & Rochira, V. (2017). Aging and sex hormones in males. Virulence, 8(5), 545–570. https://doi.org/10.1080/21505594.2016.1259053.

The third approach entails exploring the potential roles of estrogen, as well as its absence, in AD pathology. Sex steroids, such as estrogens, exert their effects through receptors found in mitochondria, along synaptic membranes, and within cell nuclei. While women of reproductive age possess higher levels of estrogens than men, these levels rapidly decline during menopause, reaching levels lower than those in age-matched men.^14^ Figure 2 illustrates the overall lifespan levels, highlighting the sharp decrease in sex hormones in women compared to a gradual decline in men. While menopause is a natural process of aging, the sharp decrease in estrogen has been associated with many health risks, such as osteoporosis.^22^ Studies have shown that cognitive task performances are diminished in postmenopausal women compared to premenopausal counterparts.^23,24^ Given recent evidence suggesting that estrogens have several neuroprotective properties, the sharp decline in estrogen during menopause could result in substantially lower neuroprotection. This, in turn, might facilitate the more rapid progression of AD pathology in women compared to men. There are other publications looking at how estrogen regulates a variety of bodily functions in women, encompassing behavior, memory, cognition, sleep, mood, pain, and coordination; here we discuss estrogen briefly for the purpose of completeness.^25-28^

### Chemical Structure of Estrogen

**Figure 3.**
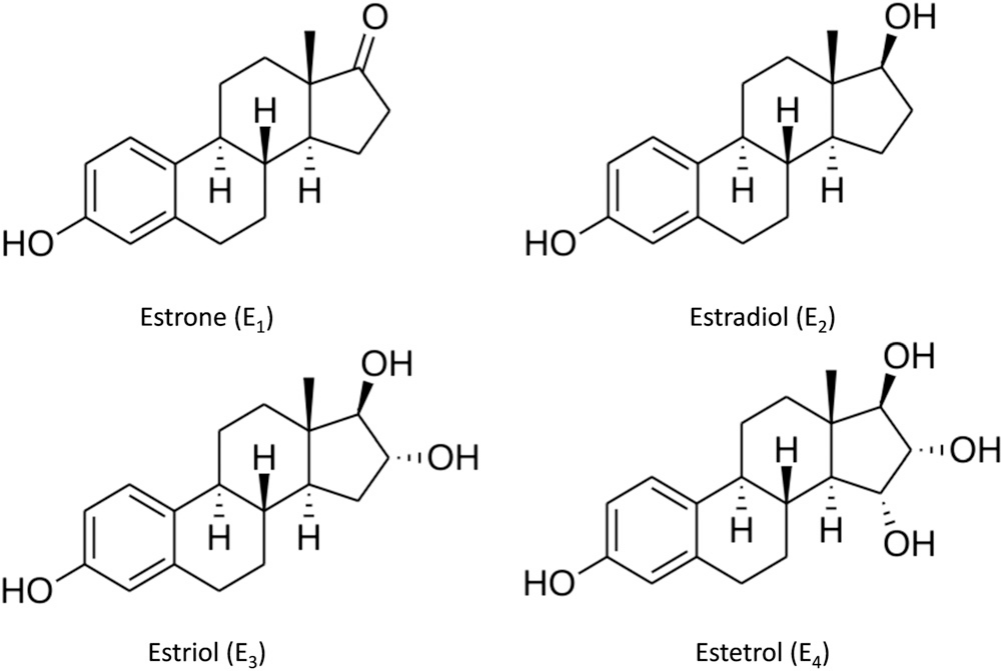
Chemical structures of estrogen. They exert their effects in several ways and play important roles in many biological functions including, but not limited to, in reproduction and the menstrual cycle, breast cancers, in osteoarthritis, in heart disease, in multiple sclerosis, in appetite and eating behavior, fat metabolism, schizophrenia, autoimmunity, and auditory and visual processing.

Estrogens belong to a class of sex hormones derived from cholesterol, and they encompass four distinct types, each named according to the number of hydroxyl groups they contain (Figure 3). Among these, estradiol (E2) is the most prevalent in humans. Estrogens are synthesized in various cells, with primary production occurring in the ovaries of females and the testes of males.^29^ While estrogens are typically associated with female-specific physiological processes such as sex organ development, menstruation, and gestation, they play integral roles in numerous pathways applicable to both sexes including insulin sensitivity and adipose tissue regulation.^30^

### Neuroprotective Properties of Estrogens

Several studies, both murine and human, have documented the neuroprotective properties of estrogens. E2 in murine has a potential role in demyelinating diseases and has been shown to regulate myelin formation.^31^ Estrogen receptors (ERs) are widely distributed in the murine central nervous system and are concentrated in parts of the brain that are involved in memory and executive function.^31^ For example, the ER isoform, ER_β_, has been shown to be found mostly in the rat cerebral cortex and hippocampus, whereas ER_α_ signaling is largely localized in magnocellular cholinergic neurons of the basal forebrain.^32^ Research has identified that E2 can induce a trophic effect crucial for memory and executive functions in the basal forebrain and the hippocampus.^33,34^ In preclinical studies, E2 replacement in ovariectomized female mice and estrogen hormone therapy in male mice have been shown to inhibit the formation of pathological human tau (hTau) by preventing amyloid-β 42 (Aβ-42) mediated hyperphosphorylation.^35^ These findings suggest that estrogen replacement therapy could potentially serve as a treatment option to slow the progression of AD pathology. In addition, an in vitro study determined that E2 can inhibit the neurotoxic properties of Aβ-42 through alternative pathways. Aβ-42-induced neurotoxicity is mediated by Thioredoxin-interacting protein (TXNIP) interactions that reduce Thioredoxin levels, which subsequently lead to increased production of reactive oxidative species and cell death. TXNIP levels were found to be highest in the hippocampus of female AD mice, and in vitro experiments demonstrated that E2 could partially reduce TXNIP levels.^36^

In addition, estrogens have been found to mediate neurotransmitter interactions in the prefrontal cortex, which is an important region mediating attention, memory and executive functions.^37^ Estrogen has also been implicated in the regulation of APOE gene transcription. The APOE gene encodes apolipoprotein E, a cholesterol transport protein. The e4 allele of this gene is a known risk factor for AD associated with Aβ-42 deposition at an earlier age.^1^ Research has revealed that estrogen plays a role in upregulating the APOE gene.^2^ Hence, a sharp decline in estrogen levels, as observed following menopause, could elevate the risk for Aβ-42 deposition. However, some studies do not find the mutation status correlating with sex or Aβ-42 load.^2,6,38^ One study noted that despite similar levels of Aβ-42 deposition in APOE-e4 men and women, women experienced a greater rate of cognitive decline than men. This suggests the possibility of an alternative pathway that makes women more susceptible to the progression of AD-related neuropathology.^38,39^

Additional studies have established that molecular mechanisms link the presence of estrogen to cognitive decline through mitochondrial function. For example, proposed models have demonstrated that hTau can be regulated in the presence of supplemental estrogen in mice following the removal of one of their ovaries.^40^ Additionally, critique of extant literature emphasized that mitochondrial dysfunction has been consistently linked to neurodegenerative symptoms.^40^ It was theorized that the abundance of E2 promotes brain mitochondrial function and prevents this outcome in young and healthy individuals through such mechanisms as gene transcription, aerobic glycolysis, mitochondrial communication, and related activity in other organ systems.^41^

Recent studies have also delved into how estrogen levels are related to brain volume in women. In a smaller study, women who underwent bilateral ovariectomy before 50 years of age were studied an average of 19 years later (between ages 62-68 years) and exhibited significantly lower amygdala volumes compared to healthy controls.^42^ There was also a decrease in the parahippocampal-entorhinal cortex area thickness. Although brief neurocognitive testing did not differ, there was a higher incidence of MCI (13%) in the ovariectomy sample compared to controls (5%). Interestingly, the clinical sample was treated for a median of 10 years with estrogen replacement, but this may not have been sufficient to offset the anatomical changes detected. Further follow-up evaluations are needed to clarify the risk for progression of symptoms.

### Ferroptosis/Oxidative Stress and Estrogen

Females are more vulnerable to neurodegeneration caused by the ferroptosis pathway due to postmenopausal loss of estrogen. Ferroptosis is a distinct type of non-apoptotic, iron-dependent cell death that is regulated in humans. One essential pathological component of neurodegeneration is iron-induced oxidative stress.^43^ In postmenopausal women, iron builds up in the brain, liver, bone, and other organs to varying degrees.^44^ Iron plays an essential role in enzymatic reactions when it is under control, as it is necessary for both oxidation and reduction processes.^45^ When uncontrolled, iron can readily transfer electrons in the Fenton reaction, producing radicals such as hydroxyl or hydroperoxyl, damaging DNA, proteins, and lipids, and ultimately resulting in cell death. The build-up of iron-mediated lipid peroxidation causes ferroptosis. Ferroptosis would result from either reduced glutathione peroxidase 4 (GPX4) activity or glutathione (GSH) depletion, which would also raise reactive oxygen species (ROS) levels and reduce lipid peroxide metabolism. The GSH-GPX4 pathway, serotransferrin-mediated iron uptake, unsaturated fatty acid-mediated lipid peroxidation, and the cholesterol synthesis-related mevalonate pathway are just a few of the intricate regulatory networks that are involved in the molecular mechanism of ferroptosis.

Because of its higher energy consumption, higher lipid content, and lower tolerance to ROS, the brain is theoretically more vulnerable compared to other organs to iron accumulation and subsequent increased oxidative stress and oxidative damage.^46^ Lower memory scores have reportedly been linked to smaller hippocampal volumes and higher regional iron concentrations.^47^ The hippocampal formation is not the only component in memory formation; however, localized injury or dysfunction can impair the formation and maintenance of various types of memory related to spatial navigation, object and conspecific recognition.^48^ It is postulated that marked ferroptosis localization and memory impairment result from iron accumulation in the hippocampi of female rats undergoing ovariectomy. E2 provided in vivo enhanced memory in female rats following ovariectomy and promoted hippocampal neuroprotection through ferroptosis resistance. Empirical investigations revealed that in primary cultured hippocampal cells treated with erastin, E2 stabilized the potential of the mitochondrial membrane and inhibited the production of ROS and superoxide damage. Following E2 therapy in vivo, these effects were accompanied by higher GSH levels and lower levels of malondialdehyde, a byproduct of lipid peroxidation. Human studies could provide additional insight into how the effects of E2 on ferroptosis suppression might help postmenopausal women with the replacement of lost estrogen, thereby potentially reducing the risk of AD pathology.

### Estrogen Replacement Therapy in Women

Given the compelling data that point to a lack of estrogen exacerbating neurodegeneration, a logical implication would be to apply this knowledge by initiating hormone replacement therapy (HRT) in estrogen-deficient women.^49^ This treatment would not be unusual as HRT is already used clinically for the management of menopausal symptoms by administering estrogen with or without progesterone. While this therapy has been proven to reduce menopausal symptoms, current guidelines advise against long-term estrogen use beyond 10 years of menopause due to the increased risk of coronary artery disease, cerebrovascular accidents, and deep vein thromboses.^50,51^

While HRT for AD prevention has been performed in several studies, the results have yielded mixed outcomes.^52^ Pike et al. (2009) have reviewed the findings concerning hormone replacement therapy and the role of estrogen as neuroprotective molecules up until the late 2000s. While some studies concluded that estrogen reduces the risk of AD, other studies report no significant change and even worse health outcomes from HRT. Due to these conflicting data, HRT is currently not employed clinically for AD prevention.

The cause for this lack of replicability is currently under investigation. While common confounding variables, such as dosage, treatment duration, prior HRT usage, and potential mediating pathways need consideration, one recurring point of interest is the age at which therapy is initiated. This suggests that there may be an optimal time to begin estrogen replacement to prevent AD, which is more favorable as HRT begins at a younger age. This is based on the data of HRT trials demonstrating better health outcomes for women who were started on HRT at an earlier age than women who started HRT much later.^14,41^ The theory is that healthier cells that are just beginning menopause react more positively to estrogen as opposed to cells that have acclimated to the post-menopausal physiological changes and interact negatively with estrogen. If this theory gains additional support, it would suggest that further study of early HRT in women who begin menopause and are at risk of developing AD should be underetaken.

However, this approach may not be feasible for all women, as not everyone benefits from HRT. This indicates the presence of other non-menopausal variables that must be considered, and that estrogen alone may not be beneficial for all women. Therefore, it becomes essential to identify these additional variables and determine which women would derive the most benefit from HRT. Given the latency period of approximately 20 years between the onset of neurodegenerative pathology and the manifestation of disease-related symptoms,^1^ establishing a reliable method to identify AD during this latency period is crucial. Detecting women at risk of developing AD or in the early stages would enable more personalized approaches to mitigate potential adverse effects observed in previous studies. One potential avenue involves leveraging the relationship between estrogen, olfaction, sleep, the glymphatic system, and cognition.

### Pharmacokinetics of Estrogen Used in HRT

Depending on the types of estrogen used and the route of administration, the pharmacokinetic properties of HRT may vary. There are different estrogen delivery methods, for instance transdermal, intramuscular, intravenous, percutaneous, and oral. Consequently, the type of estrogen used and its mode of administration affect the precise concentrations of estrogen needed to alleviate postmenopausal symptoms.^53^ Different estrogen products result in different systemic concentrations of estradiol and estrone, as well as different ratios of estrone to estradiol. Estradiol and estrone levels produced by conjugated equine estrogens (CEE) at clinically effective doses are similar to those of a typical menstrual cycle.^54^ Estradiol concentrations are higher when administered transdermally or subcutaneously than when taken orally.^53^ High systemic levels of estradiol, comparable to those of transdermal products, are achieved by intravaginal products.^55^ Higher estradiol concentrations are produced by estradiol vaginal creams, while higher estrone concentrations are produced by CEE vaginal creams. Additionally, higher estradiol and estrone concentrations were found with intravaginal CEE than with oral CEE.^56^

While conjugated and unconjugated estrogens are easily absorbed from the gastrointestinal tract, oral estrogens have low bioavailability. Certain unconjugated estrogens, such as equilin vs equilin sulfate, may be absorbed relatively quickly compared to conjugated estrogens. Once absorbed, the unconjugated estrogens are rapidly conjugated in the liver.^57^ In contrast to estrone, which is also inactive, most equilin and estrone circulate in their sulfated inactive form.^58^ However, the half-life of estrone sulfate is longer.^59^ The fact that unconjugated estrone and equilin gradually accumulate in the blood following CEE administration, therefore, suggests that the pool of estrogen sulfate resulting from oral CEE or estradiol functions as a hormonally inert estradiol reservoir that can be gradually converted to active hormone.^57,60^ Following CEE administration, the presence of an inactive hormone pool may result in a longer-lasting response and a more gradual onset of estrogen action, protecting against abrupt estrogenic stimulation.^61,62^ Due to first-pass metabolism in the liver and gut, oral estrogens cause a noticeable hepatic reaction. For instance, oral estrogens stimulate the hepatic production of corticosteroid-binding protein and sex hormone-binding globulin (SHBG). Greater increases in high-density lipoprotein cholesterol and smaller decreases in low-density lipoprotein cholesterol, lipoprotein (a), and insulin resistance are among the advantages that can result from the first-pass effect. However, adverse outcomes are also observed, including elevations in triglycerides and overactivation of coagulation pathways. Parenteral estrogens lead to lower concentrations of estrogen metabolites and higher concentrations of the original compound because transdermal, percutaneous, and intravaginal estrogen products avoid metabolism in the gut and liver. Eliminating first-pass metabolism reduces the effects of estrogen on the liver. Transdermal administration of estrogens does not result in an increase in SHBG, whereas oral administration does.^63^ Nevertheless, avoiding the liver may not always be advantageous because lipid profiles may not be optimally improved by the reduced action on the liver.

## The Role of Estrogen in AD-Related Olfactory Impairment

As previously mentioned, olfactory dysfunction has been recognized as an early sign of neurodegenerative disorders.^64,65^ Its high prevalence, early onset, and the availability of sensitive olfactory tests have heightened interest in establishing detection of olfactory dysfunction as an early marker of neurodegeneration.^66-69^ Impaired smell with an associated decrease in olfactory ability has been proposed as an initial indicator of classical neurodegenerative disorders such as AD and Parkinson’s disease.^70-72^ The use of olfactory dysfunction as a biomarker for neurodegenerative diseases is useful in the characterization of the prodromal stages identifying early diagnostic strategies, facilitating differential diagnoses, and predicting clinical outcomes. In general, focusing on olfactory function can help improve the success of early intervention and therapeutic strategies.^73^ Since olfactory deficits are known precursors to AD,^74,75^ olfactory function could be used to gauge the risk of developing AD.

Estrogen has been linked to olfactory performance.^76^ In oophorectomized mice, olfactory deficits were prevented when treated with E2 before and after the introduction of Aβ–42 peptide into the brain.^76^ Another study showed that E2 treatment in oophorectomized mice can lead to increased olfactory epithelium width.^77^ This effect was postulated to be due to increased basal cell proliferation, protection of mature neuronal death, and increased axonal growth, all being related to neuroprotection.^77^ In humans, an improved sense of smell was observed and found to be weakly significant in women undergoing HRT.^14,15^

Estrogens play a direct role in olfactory processing in mammals and the preservation of olfactory memories, a process that likely begins at the olfactory bulb level.^78,79^ Circulating estrogens have a positive impact on the development, morphology, and synaptogenesis of olfactory sensory neurons (OSNs). Estrogen signaling is crucial for the development of both OSNs and sustentacular cells. Studies have documented the protective role of estrogen against 3-methylindole-induced olfactory loss.^80^

A recent fMRI study revealed lower activation of the primary olfactory cortex and hippocampus in women than in men in a cohort between the ages of 50 and 60.^81^ In the same study, they showed that older men have steeper decline in olfactory fMRI activation than women, with an intersection taking place at around age 70. While this may seem puzzling at first, there are a few theories to consider. One potential theory is that the baseline olfactory pathways in women are more efficient than in men, requiring less brain activation to process stimuli compared to men. Alternatively, the olfactory activation in women could be limited due to the sharp decline in estrogen following menopause as opposed to the gradual decline observed in men. This highlights the need for additional olfactory fMRI studies targeting perimenopausal women to ascertain if there is a gradual or sharp loss in olfactory activation during menopause.

## The Role of Estrogen in Sleep

An intriguing observation is that sleep disturbances usually accompany Alzheimer’s disease and are known to increase the risk of developing dementia.^82,83^ Women have been observed to be disproportionally impacted by sleep problems compared to men.^84^ Sleep difficulties frequently start during the menopausal transition. For example, vasomotor symptoms, restless leg syndrome (RLS), nocturnal awakenings, and sleep apnea are common complaints among women transitioning into menopause. Self-reported sleep problems can range from 40% to 56% of post-menopausal women compared to 31% of pre-menopausal women.^85,86^ The Study of Women’s Health, which collected data from more than 3000 women over an 8-year period, revealed that the most common type of sleep issue is night waking.^87^

As for the RLS disparity, many hypotheses have been proposed. Theories include iron deficiency secondary to pregnancy, persistently high levels of estrogen during pregnancy, and the decline of estrogen and melatonin during menopause.^86^ Regarding the role of estrogen in the etiopathogenesis of RLS, Saltzman et al. hypothesized that estrogen may affect the catechol-O-methyltransferase (COMT) enzyme, inhibiting the dopaminergic system, resulting in increased dopamine catabolism.^88,89^

A study focused on the connections between E2, follicle stimulating hormone (FSH), and sleep disorders showed that sleep disturbances are aligned with changes in hormone levels associated with menopause. In particular, decreases in serum E2 levels were linked to reports of difficulty falling asleep as well as difficulty staying asleep, whereas increases in serum FSH levels were linked to reports of difficulty staying asleep.^90^ A double-blinded study reported improvement in time to fall asleep and a reduction of night awakenings in menopausal women who used transdermal E2 with progesterone.^84^ These results remained significant when correcting for improvement of vasomotor and depressive symptoms when initiating HRT, suggesting that E2 may contribute to additional sleep-related pathways.^84^

## The Glymphatic System and Potential Mechanisms of Sleep Impairment

While these studies show that estrogen changes can be a contributing factor to sleep disturbances, mechanisms of sleep impairments associated with Alzheimer’s pathology is not fully understood. A potential pathway to consider is though the glymphatic system, which clears waste via cerebrospinal fluid (CSF) circulation.

The glymphatic system is most active during sleep due to glial cells contracting during rest,^91^ causing the extracellular space to expand and speed up the process by which waste and other metabolites, including Aβ, are cleared from the brain. In murine models, the clearance of pathological solutes such as Aβ was twice as quick while the animals were asleep than awake.^92^ Additionally, when compared to mice under anesthesia, photon imaging studies have revealed a 90% reduction in glymphatic activity in awake mice.^92^ An in vivo study in humans demonstrated the use of blood oxygen level-dependent functional magnetic resonance imaging, electroencephalogram (EEG), and CSF measurements to identify the sleep state with the highest levels of brain activity. They discovered that CSF flow had a small-amplitude rhythm during wakefulness, peaking at about 0.25 min^−1^, whereas during sleep, large oscillations occurred every 20 seconds, resulting in a noticeably higher inflow of CSF than during the day.^93^

These changes in sleep patterns have been observed to impact brain functionality, where it is readily observed in olfactory regions of the brain. The piriform cortex loses its odor sensitivity during slow-wave sleep (SWS) and instead exhibits sharp-wave activity, resembling that of the hippocampal formation. SWS is recognized as playing a significant role in brain plasticity, including the modulation of synaptic connectivity and the survival of olfactory bulb neurons.^94,95^ Furthermore, sleep has been observed to be essential for odor memory and individual odor perception.^96,97^ Additionally, when compared to wakeful periods, SWS has greater functional connectivity between the piriform cortex and other cortical and limbic regions.^98^ During sleep, odor memory consolidation may strengthen the associations between odors and their previously established contextual or hedonic cues. These findings imply that sleep affects the potency and accuracy of odor memory formation. As a result, the strength and precision of odor memory can be affected by poor sleeping habits, disrupting the normal sleep-regulated activity in the olfactory cortex.

## Discussion and Conclusion

Based on current sampling and statistics, women are at an increased risk of developing AD compared to men. Deciphering the cause of this sex disparity, particularly in relation to estrogen, may reveal new information about AD pathophysiology and help advance identification of risk factors, diagnostic tools and treatment options for all genders. A patient’s clinical history, symptoms, the presence of tau deposition and β-amyloid plaques (as demonstrated by spinal fluid measurements and positron emission tomography imaging of tau deposits or β-amyloid plaques) and post-mortem investigations are key information sources currently being used to diagnose and confirm AD. Many of the pathological alterations observed in brain tissue begin to occur years before the onset of clinical symptoms, and deciphering what role estrogen variations may play in these processes is an important scientific question for future research. While menopause and estrogen levels likely are important elements in the elevated incidence rates of AD among women, in this review we highlighted potential roles of estrogen in olfaction, sleep, and glymphatic functionality that may provide additional perspectives on the sex disparity observed in AD.

Women have a steep decline in estrogen during menopause while estrogen gradually declines in men. This may lead to different patterns of disruption to several neuroprotective properties of estrogen. For example, reduced glucose metabolism in FDG-PET has been observed in postmenopausal women in regions susceptible to AD pathology, including the lateral temporal cortices, frontal, parietal, medial, and posterior cingulate/precuneus regions bilaterally.^99^ Early menopausal surgery age has been linked to a higher risk of cognitive decline and Alzheimer’s disease-related pathology in women.^100^ Hormone replacement therapy after menopause has been shown in some clinical trials to lower the risk of AD.^101^ Transdermal E2 treatment has been found to decrease β-amyloid deposition in women who had recently gone through menopause,^102^ suggesting that E2 may have an effect on the formation of β-amyloid plaques.

Reduced expression of genes linked to amyloid processing is a consequence of lower E2 levels, as preclinical research has shown.^103^ In vitro investigations have shown that amyloid is preferentially processed to the soluble form upon E2 stimulation^104,105^ Additionally, E2 controls the activity of β-amyloid degrading enzymes, such as neprylisin^106,107^ and metalloproteases^108^ which may lessen the development of β-amyloid plaques in vivo. Subsequent in vivo research revealed that E2 therapy can pharmacologically attenuate tau hyperphosphorylation^109^ and decrease tau phosphorylation.^110^ This decrease in phosphorylation might also contribute to a reduction in the pathology linked to AD.

AD can be thought of as causing dysregulation in different biological systems. For instance, as the disease advances, the basal forebrain cholinergic system deteriorates,^111^ although E2 therapy seems to help maintain the cholinergic system.^112,113^ Similar changes occur in the mitochondrial bioenergetic system in Alzheimer’s disease, leading to elevated ketone metabolism and reactive oxygen species generation. This process is also observed preclinically and clinically after menopause, and estrogen can mitigate these changes.^103^

**Figure 4.**
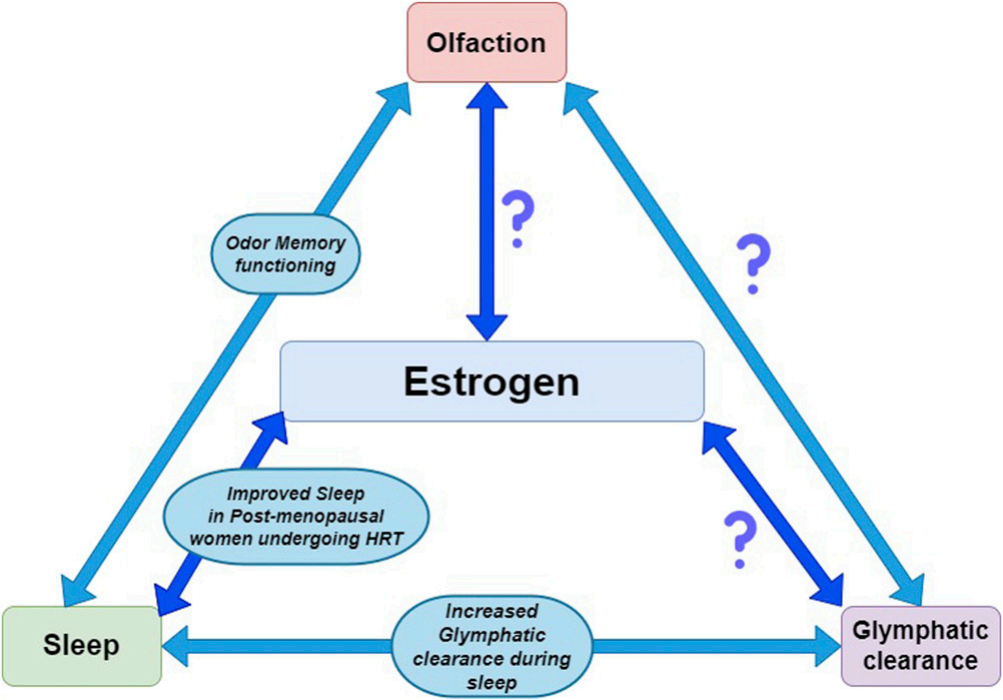
Known and unknown relationships between estrogen, sleep, olfaction, and the glymphatic clearance system. Investigating other biological functions that are impacted by estrogen as well as their associations with neuropathology is warranted. This is to understand estrogen mediated neuroprotection and to determine who would best benefit from hormone replacement therapy (HRT).

Despite encouraging findings, HRT in women has resulted in inconclusive and sometimes contradictory findings in regard to AD incidence rate. The data suggest the involvement of additional compounds and/or alternative pathways, as estrogen alone may be beneficial for some women, but not all. The investigation of other variables that are impacted by menopause as well as their associations with neurobiological health, is necessary in order to fully understand the role of estrogen in neuroprotection and to determine who would best benefit from HRT. Based on available research, we hypothesize that several variables need to be further investigated in relationship to estrogen and AD, including olfaction, sleep, and the glymphatic system as described in Figure 4.

Humans have complex olfactory pathways tightly connected to memory systems that are impacted by neurodegeneration.^114^ The olfactory system is affected early in patients with AD and PD.^16^ In particular, odor identification ability in MCI and Alzheimer’s patients is significantly lower than that of normal controls, and this ability worsens as patients progress from MCI to AD.

Similarly, there are increased sleep disorders and associated symptoms including vasomotor symptoms, restless legs, night awakenings, and apneas during menopause. The main risk factors for insomnia is sex and age^115^; compared to men, women are 40% more likely to experience insomnia over the course of their lifetime,^116,117^ and this raises the possibility that a woman’s physiology plays a major role in insomnia. Insufficient sleep can increase the risk for developing neurodegenerative diseases like AD. Moreover, compared to age-matched males, alterations in ovarian steroid production, such as those that take place during puberty and menopausal transition, are linked to a higher frequency of insomnia and poor sleep.^118,119^

With higher frequencies of sleep problems, the menopausal transition is a well-known indicator of poor sleep.^120^ As estrogen replacement therapy is effective in reducing sleep disruptions during this period, it is likely that the loss of ovarian estradiol production is responsible for the disturbances in sleep.^121,122^ The understanding of the relationships between ovarian steroids and women’s sleep is largely lacking due to the dearth of studies examining sleep in reproductive-age women, inconsistent experimental paradigms, and small sample sizes of existing studies.

In general, the menopausal transition is characterized by unpredictable fluctuations in estrogen levels that eventually decrease over time. As mentioned earlier, a prevalent sign of menopause transition is the perception of disturbed and poor sleep.^123^ This low estrogen-mediated poor sleep can potentially contribute to the progression toward neurodegeneration; putting age-matched women at a higher risk for developing AD pathology. Objective sleep metrics, however, fail to capture this decline in sleep quality.^124^ There is a significant knowledge gap regarding the discrepancy between objectively measured sleep metrics and subjective sleep complaints in postmenopausal and perimenopausal women. The severity of menopausal symptoms may very well determine how much sleep disturbance occurs during menopause. The menopausal transition is accompanied by hot flashes, which affect 75%-85% of women which is clearly linked to sleep disturbances.^123,125^ Hormone therapy has been shown to enhance the quality of sleep suggesting that ovarian steroids and estrogens play a role in the sleep cycle.^126^

Research on the effects of sex steroids on sleep, in both men and women, has produced mixed results. Beyond clinical observations that sex steroids impact sleep behaviors, there is still a significant knowledge gap regarding mechanisms by which sex steroids affect the sleep circuitry. Finding relationships between estrogen fluctuations and the sleep circuitry would prove valuable in further deciphering the sex disparity of AD.

As discussed above, many studies have shown that estrogen plays a critical role in the occurrence and severity of sleep disorder symptoms. Poor sleep has been associated with an increased risk of AD. Investigating whether menopause-associated sleep difficulties increase the risk of developing AD is a promising avenue for future research.

A related hypothesis is that the relationship between poor sleep and AD risk is via the glymphatic system. The glymphatic system and sleep are closely related. Menopausal women who experience poor sleep may also suffer from impaired glymphatic functionality. This would cause inadequate neurotoxin clearance, resulting in an elevated risk of developing or worsening neurodegenerative conditions.^91,127^ The evaluation of risk factors and treating sleep disorders that are potentially linked to estrogen instability may be beneficial for clearing toxic aggregates, potentially ameliorating symptoms of neurodegeneration. In addition, we hypothesize that these toxins, unable otherwise to cross the blood-brain barrier, may induce inflammation in the olfactory mucosa, olfactory bulb, and entorhinal cortex first before spreading to other brain regions, resulting in preclinical olfactory dysfunction.^127^

A reliable means of detecting changes indicative of possible AD pathology buildup during the latency period, prior to the onset of neuropathology and clinical symptoms, will be useful in verifying whether estrogen plays a pivotal role in the observed sex disparity in AD. Despite extensive research into the neuroprotective properties of estrogen, there remains a clear gap in current understanding regarding the impact of estrogen loss due to menopause on cognition, particularly in relation to AD.^10^

Lower longevity of men, meaning that men might be passing away from other causes before AD symptoms become clinically evident, has been suggested as a reason for the observed sex disparity in AD. This is a debatable idea that could be tested by assessing post-mortem AD biomarkers in large samples of men to determine if there is neurobiological evidence of AD at the same rate as women. An alternative means of testing this idea would be through the use of earlier diagnostic tools; for instance, focusing on olfactory deficits that are linked to early AD pathophysiology.^16^

An intriguing and potentially promising avenue for further research suggested in this review article can involve a combination of human olfactory functional magnetic resonance imaging (fMRI) with an assessment of glymphatic system functionality. As previously mentioned, a study has already observed lower olfactory fMRI activation in women aged 50 to 60 compared to men. However, older men exhibited a sharper decline in olfactory fMRI activation, resulting in lower overall fMRI activation for men above 70 years old. Currently, there is a dearth of literature investigating olfactory fMRI activation in women before, during, and after menopause.

**Figure 5.**
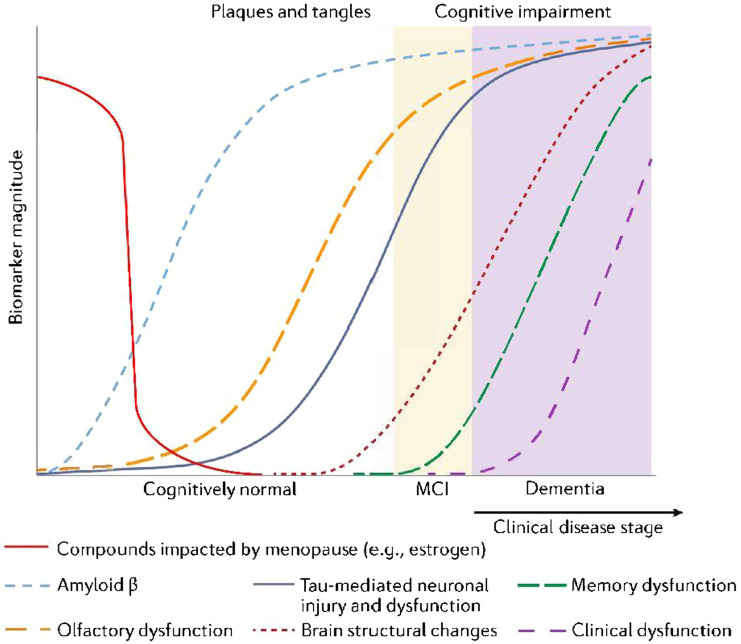
Theoretical graph representing proposed correlations between menopause and the onset of neuropathology that may result in AD. Higher levels of estrogen results in lower levels of human Tau and Aβ-42, highlighting potential neuroprotective properties of estrogen. Olfactory function is also expected to be unimpaired when estrogen levels are high.

If the Martinez et al. (2017) study is expanded using a cohort of perimenopausal women, it would allow for further investigation into whether there is a significant decline in olfaction-related fMRI activation that may be proportional to estrogen levels during menopause.^81^ An interesting possibility for the olfactory fMRI task would be in the domain of olfactory identification, which is significantly affected in AD.^16^ Such studies should include ≥25 subjects per group following standard fMRI methodology.^128^ It should be noted that studying the effects of estrogen in humans is challenging. There are many confounding factors which need to be controlled for in such studies including adiposity, diet, diet supplements, use of SERMS, vaginal creams etc. In addition, there are the confounding risk factors for Alzheimer’s disease to be considered, as mentioned in this review. Nevertheless, if there are significant correlations, this would provide additional support for the hypothesis of the neuroprotective properties of estrogen, as visualized in Figure 5. This also shows where olfaction and estrogen may interact during the AD cascade—an important perspective put forth in this review article. Furthermore, conducting longitudinal studies could help determine whether changes in estrogen levels and related biological functions such as olfaction (in terms of fMRI activation), sleep and, glymphatic function predict cognitive impairment and progression to AD. These can potentially bolster targets for intervention and prevention.
